# Tissue Engineering Scaffolds Fabricated in Dissolvable 3D-Printed Molds for Patient-Specific Craniofacial Bone Regeneration

**DOI:** 10.3390/jfb9030046

**Published:** 2018-07-24

**Authors:** Angela Alarcon de la Lastra, Katherine R. Hixon, Lavanya Aryan, Amanda N. Banks, Alexander Y. Lin, Andrew F. Hall, Scott A. Sell

**Affiliations:** 1 Department of Biomedical Engineering, Saint Louis University, St. Louis, MO 63103, USA; angela.alarcondelalastra@slu.edu (A.A.d.l.L.); katherine.hixon@slu.edu (K.R.H.); lavanya.aryan@slu.edu (L.A.), amanda.banks@slu.edu (A.N.B.); andy.hall@slu.edu (A.F.H.); 2 Department of Surgery, Saint Louis University, St. Louis, MO 63104, USA; alexander.lin@health.slu.edu

**Keywords:** cryogel, hydrogel, 3D printing, patient-specific, craniofacial defects, bone regeneration, tissue engineering

## Abstract

The current gold standard treatment for oral clefts is autologous bone grafting. This treatment, however, presents another wound site for the patient, greater discomfort, and pediatric patients have less bone mass for bone grafting. A potential alternative treatment is the use of tissue engineered scaffolds. Hydrogels are well characterized nanoporous scaffolds and cryogels are mechanically durable, macroporous, sponge-like scaffolds. However, there has been limited research on these scaffolds for cleft craniofacial defects. 3D-printed molds can be combined with cryogel/hydrogel fabrication to create patient-specific tissue engineered scaffolds. By combining 3D-printing technology and scaffold fabrication, we were able to create scaffolds with the geometry of three cleft craniofacial defects. The scaffolds were then characterized to assess the effect of the mold on their physical properties. While the scaffolds were able to completely fill the mold, creating the desired geometry, the overall volumes were smaller than expected. The cryogels possessed porosities ranging from 79.7% to 87.2% and high interconnectivity. Additionally, the cryogels swelled from 400% to almost 1500% of their original dry weight while the hydrogel swelling did not reach 500%, demonstrating the ability to fill a defect site. Overall, despite the complex geometry, the cryogel scaffolds displayed ideal properties for bone reconstruction.

## 1. Introduction

Orofacial defects are the most prevalent craniofacial birth defect, with oral clefts affecting approximately 1 in 700 live births [1]. Typically, the palate bone separates the mouth from the nose; however, with cleft palate a part of this bone is missing [2]. These defects negatively affect the quality of life of the patient, specifically the facial growth, function, dental development, and aesthetics [3]. In addition to this, the cost of treatments and rehabilitation for patients with oral clefts can reach up to $100,000 [1]. Currently, the standard treatment is an autologous bone graft taken from the iliac crest to reunite the cleft, resulting in another wound site, donor site morbidity, and greater discomfort for the patient [3,4,5]. In addition, pediatric patients have less bone mass and require an invasive procedure. Furthermore, these craniofacial defects are complex and three dimensional (3D) which presents more complications, as bone grafts lack site specific geometry of the patient [6].

Currently, there is considerable work being done to develop off-the-shelf tissue engineering scaffolds. Tissue-engineered scaffolds can provide a framework for cell proliferation, migration, and attachment and, due to these characteristics, are emerging as popular treatments for bone regeneration and wound healing. Additionally, as scaffolds provide a temporary framework for regeneration, the mechanical properties should support tissue growth, where the scaffold degrades as new tissue is formed [4,7]. There are various methods for creating porous scaffolds including porogon leaching and gas foaming [8,9]; however, this paper will focus solely on hydrogels and cryogels fabricated through chemical crosslinking. Hydrogels are a well-characterized scaffold in tissue engineering formed through the physical or chemical crosslinking of a polymer/monomer aqueous solution, resulting in the formation of a nanoporous gel structure composed of 99.9% water. These scaffolds have been investigated for the regeneration of bone, cartilage, neural, and other tissues [10,11,12,13]. Similarly, cryogels are also produced through the crosslinking of a polymer/monomer aqueous solution, but are immediately placed at sub-zero temperatures for formation [14,15]. This results in ice crystals forming and expanding and, when thawed, the ice crystals melt out, leaving the interconnected pore structure. This critical difference in fabrication protocol forms scaffolds with an interconnected, macroporous, and sponge-like structure possessing a high elastic modulus and the ability to remain intact without damage even after 50% compression [7,14,16]. Parameters such as freezing time and temperature, type of polymer, method of crosslinking, and cooling time can be altered to modify the cryogel for the desired application [4,7,17]. Cryogels have been investigated for a variety of tissues such as the regeneration of cartilage, corneal stroma, adipose tissue, and bone, among others [7,18,19,20,21,22]. Various in vivo studies have demonstrated the applicability of these scaffolds in cranial bone defects [23,24,25]. In one such study by Liao et al. [24], cryogels composed of gelatin, hydroxyapatite nanoparticles, and bone morphogenetic proteins, along with seeded allogenic adipose-derived stem cells, had successful bone formation when implanted in rabbit critical size calvarial defects. Histological and immunohistochemical analyses also showed that new bone was formed and there was expression of collagen 1 and osteocalcin bone-specific proteins present at the defect site. Overall, these studies demonstrated the applicability of cryogels in the tissue engineering and bone regeneration field.

3D printing (3DP) is also an emerging area of development within medicine, and tissue engineering in particular [26,27,28]. 3DP is primarily based on additive manufacturing technologies, which build 3D structures layer by layer. Both adapted industrial 3DP technologies and custom-built printers have been used. Examples include fusion deposition modeling, stereolithography, laser sintering, and inkjet printing. While 3DP technologies have been clinically deployed in craniomaxilofacial surgery, they are primarily used in the areas of models, guides, splints and implants [29].

There are three main approaches to 3DP in tissue engineering: printing live cells (bioprinting), printing acellular scaffolds, and printing molds to be filled with engineered tissue [16,28,30]. 3DP tissues have two, largely independent, resolution requirements; the micro-structure resolution, which is dependent on the spatial resolution of the printer, and the macro-structure resolution, the overall shape of the tissue volume. The micro-structure resolution is normally comprised of the in-layer (x–y) resolution and the layer height (z). While layer heights can be as low as 20 µm, in-layer resolutions range from 125 to 250 µm [28] (Some stereolithography printers have reached 70 µm). More recently, electrohydrodynamic-jetting printers have been developed with fiber sizes as low as 50 µm [31]. While this technology is promising, arbitrary 3D macro-structures have not yet been printed. Tissue macro-structures are much larger and are often designed for personalized medicine, where the tissue’s shape is derived from a 3D medical image [16,30].

Currently, using 3DP technology to print the micro-structure can impose limitations on the material used. For example, filament deposition modeling printers require the material to be melted and extruded, and ink-jet printers require a liquid material. Using 3DP to print a mold in the shape of the desired macro-structure can provide more freedom in the tissue type selection, provided a single mixture is used. Furthermore, these tissue types can have smaller micro-structures than those that can be printed by 3DP. For example, cyrogels can be manufactured, using molds, that have pore sizes less than 20 µm, and a strut thickness in the nanometer range [16].

There are currently no recent studies that have used 3DP to create dissolvable molds for scaffold fabrication for bone regeneration, except for the preliminary tests done previously in our lab [16]. Our lab has demonstrated that 3D printing technology can be used in conjunction with cryogel fabrication to create CT-derived, patient-specific molds for the fabrication of these scaffolds. However, our previous study used non-dissolvable molds that were printed in parts and glued with silicone. These parts were then pulled apart to take the scaffold out. This method is not very feasible because it would break apart the scaffolds while opening up the mold. By combining 3D printing and scaffold fabrication one is able to create scaffolds with the exact geometry of bone defects. This study will further the work previously done in our lab by investigating the use of dissolvable 3D printing mold materials to make it easier to remove the scaffold, thus alleviating post-fabrication deformation and improved fit.

In this study, the cryogels were made from chitosan and gelatin, similar to a previous study [32]. Chitosan has been shown to have excellent properties for biomedical applications such as biocompatibility, biodegradability, non-toxicity, adsorption properties, and the ability to be degraded by lysozyme [32]. Gelatin, a denatured form of collagen which is present in tissues, is also biocompatible, biodegradable, nonantigenic, and has natural cell adhesion sites [18,32]. Previously fabricated chitosan-gelatin (CG) cryogels have been shown to possess pore diameters of 30–100 μm, high mechanical integrity, and fibroblast adherence [32,33].

To date, there have been few studies that have combined cryogel scaffolds and 3DP strategies for the purpose of bone regeneration. The 3DP technology and scaffold fabrication can be combined to create patient-specific cryogels with the necessary microarchitecture for implantation. In this study, three different CT scans of children with cleft-craniofacial defects were used to 3D print molds of various materials to fabricate tissue engineering scaffolds (cryogels and hydrogels). The molds were printed from polyvinyl alcohol (PVA), acrylonitrile butadiene styrene (ABS), and high impact polystyrene (HIPS) which dissolve in water, acetone, and d-Limonene, respectively. These scaffolds were then characterized to determine pore size, swelling kinetics, porosity, mechanical integrity, and cell compatibility.

## 2. Results

### 2.1. Creation of Scaffolds of Various Geometries

**Figure 1:**
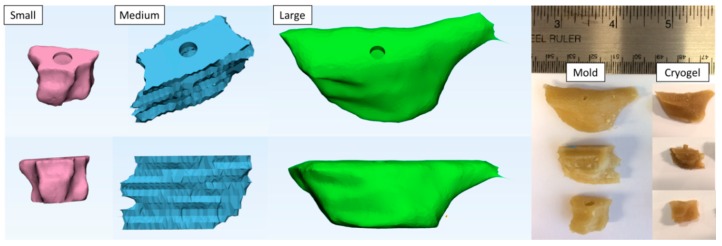
The CAD images represent the defect molds. The image on the right hand side visually compares the mold to the cryogel that was fabricated in the mold.

The defect site was isolated from each of the three patients’ maxilla and hollow molds of the three defect sites were created, as shown in Figure 1. All of the molds for these three defect sites were printed with a 1 mm thickness. The molds were printed in three materials: PVA, ABS, and HIPS. PVA and ABS molds were used to fabricate the cryogels, while PVA and HIPS were used to fabricate the hydrogels. Note that these materials were chosen based on ease of removal and effect on scaffold structure, as determined by pilot testing. Specifically, cryogels fabricated in HIPS molds were difficult to remove due to the length of time required for dissolution. Further, hydrogels formed in ABS molds were extremely fragile and fractured upon removal. The cryogels were fully formed after 24 h and the hydrogels were fully gelled after 2 h in their respective molds. PVA and ABS molds dissolved in 2–4 h, while HIPS molds dissolved in 5–8 h. The shape of the cryogels formed in PVA molds can be observed in Figure 1. As shown in the image, all cryogels were able to accurately maintain the shape of the defect site. The following results provide characterization of these scaffolds and the fit test data provides quantitative data on the defect volume to scaffold ratio.

### 2.2. Pore Analysis (SEM and µCT)

#### 2.2.1. SEM

**Figure 2:**
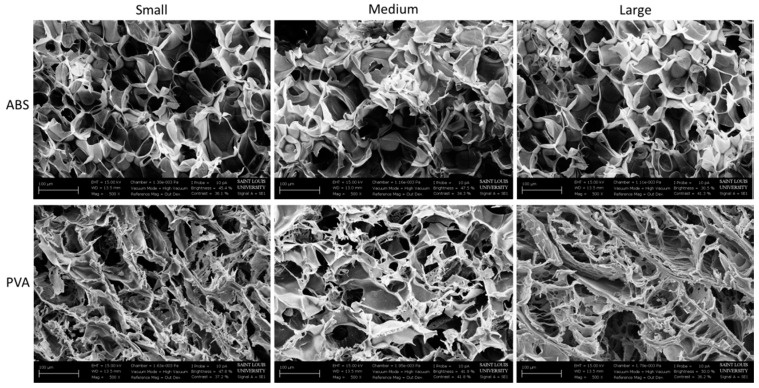
SEM images of the cryogels in the ABS and PVA molds taken at 500× for each geometry.

**Figure 3:**
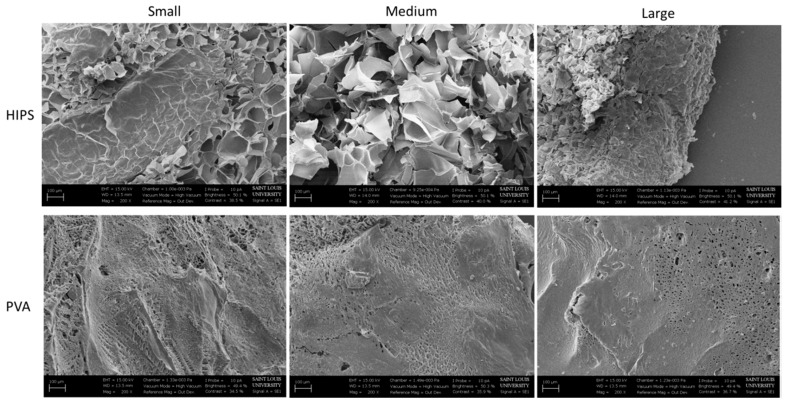
SEM images of the hydrogels in the HIPS and PVA molds taken at 200× for each geometry.

SEM images were taken at a magnification of 500× for the cryogels fabricated in the ABS and PVA dissolvable molds. SEM images provide a visual representation of the pore sizes; however, as the images are taken of dehydrated scaffolds, the pore size and structure are not representative of the hydrated scaffolds, as described previously [33]. As seen in Figure 2, the pores on the cryogels from the ABS molds appear uniform and round throughout, regardless of the mold geometry. The pores on the cryogels formed in the PVA molds appear flakier in all three geometries. The SEM images in Figure 3 were taken at 200× and demonstrate the pore sizes of the hydrogels in the HIPS and PVA molds. Due to the high water content, most of the hydrogels did not appear to possess a porous structure. Variations in this may be due to the lyophilization process. Overall, the pore sizes on the hydrogels from the HIPS molds appear larger than the pores in the hydrogels from the PVA molds. However, the hydrogels’ pore sizes appear smaller than the cryogels’ pore sizes. Due to the 2D nature of the SEM images, and inherent inaccuracy of measuring pixels in the SEM images, the images were not used for measuring mean pore sizes.

#### 2.2.2. µCT

**Figure 4:**
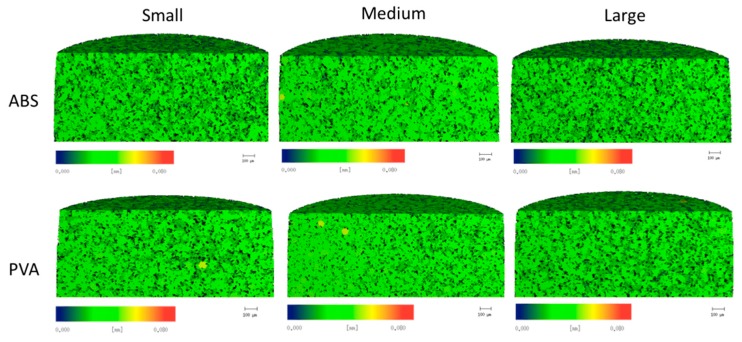
µCT scans of the cryogels in the ABS and PVA molds represent the inner pore sizes with the color bar denoting the size of the pores within the cryogel.

**Figure 5:**
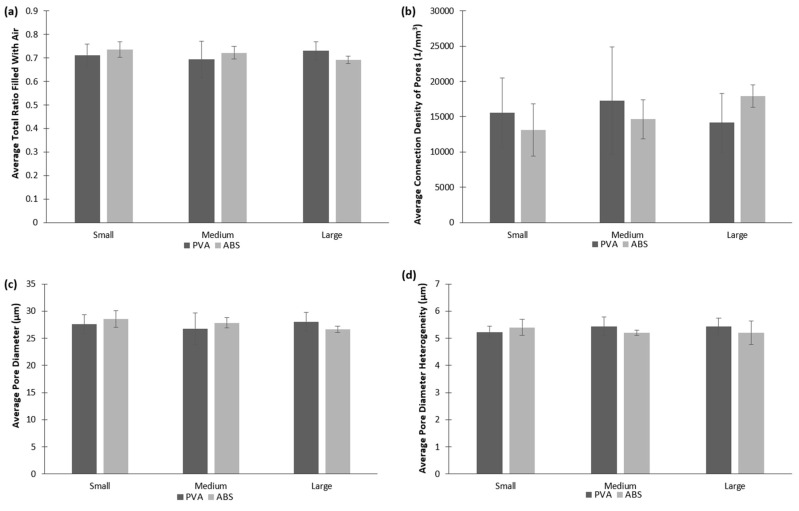
µCT scans provide quantifiable data for the cryogels’ pore sizes and interconnectivity. (**a**) represents the average ratio of air to scaffold; (**b**) represents the average connection density of the cryogels’ pores; (**c**) represents the average pore diameter; and (**d**) represents the average pore diameter heterogeneity.

µCT analysis was used to provide quantifiable data of the cryogels’ pore sizes, heterogeneity, air to scaffold ratio, and connection density. As seen in Figure 4, pore sizes appear to be uniform throughout all scaffolds, further supported by the data presented in Figure 5. No statistically significant difference in the pore sizes, heterogeneity, ratio of polymer to air, and connection density was found between any of the cryogels’ mold geometries and dissolvable materials (*p* < 0.05). The average total ratio of the air to scaffold ranged from 0.69 to 0.73. The average connection density ranged from about 13,000 1/mm^3^ to almost 18,000 1/mm^3^ with the large cryogel in ABS having the highest average connection density. The average pore diameters ranged from 26.6 to 28.6 µm for all cryogels. Lastly, the average pore diameter heterogeneity ranged from 5.2 to 5.4 µm. A cell infiltration study was done to further verify that the pore sizes and heterogeneity were appropriate for in vivo applications.

### 2.3. Porosity

**Figure 6:**
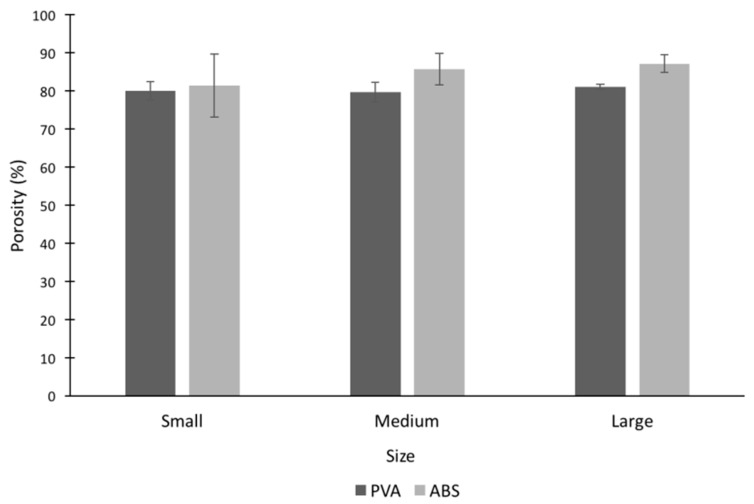
All cryogel scaffolds had porosities ranging from 79% to 87% with no statistically significant differences.

Porosity of the cryogel scaffold was measured to determine the void space in the cryogel scaffolds (Figure 6). Note that the hydrogels were not included in porosity measurements due to their nanoporous structure [7]. The cryogel scaffolds had porosities ranging from 79.7% to 87.2% with the medium size cryogel in PVA having the smallest value and the large size cryogel in ABS having the highest value. There was no statistically significant difference between any of the cryogels, regardless of geometry or mold material. These numbers closely reflect the values obtained in the µCT (Figure 5a).

### 2.4. Swelling Kinetics

**Figure 7:**
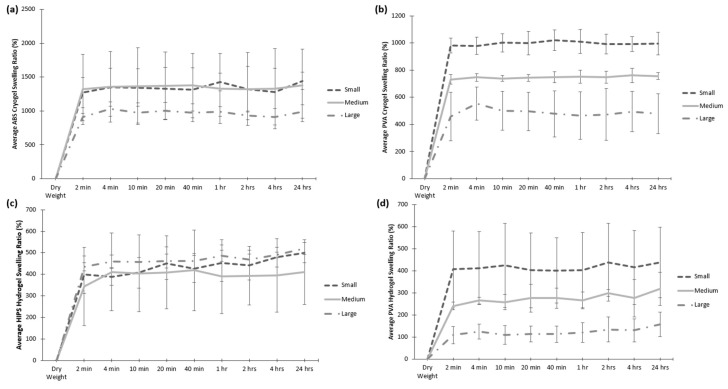
The dehydrated scaffold (denoted as dry weight) were soaked in water for a 24 h period and the dry weight was used to calculate the swelling ratio (%). This is provided for (**a**) ABS and (**b**) PVA cryogels, as well as (**c**) HIPS and (**d**) PVA hydrogels. The scaffolds fabricated in PVA molds had significantly different swelling ratios between the different shapes (*p* < 0.05), while the cryogels fabricated in ABS molds and the hydrogels fabricated in HIPS molds were similar between the different shapes.

Swelling tests were performed to determine how well the scaffolds were able to retain their shape and rehydrate to fill a bone defect site. As shown in Figure 7, all scaffolds re-swelled up to at least 100% of their original dry weight within 2 min of being submerged in water. Overall, the cryogels were able to swell from 400% to almost 1500% of their original dry weight whereas the hydrogels’ swelling ratios were mostly under 500%. There were more differences in the swelling potential between the geometries of the cryogels and hydrogels in the PVA molds than those in ABS and HIPS. In the scaffolds fabricated in PVA molds, the swelling potential decreased from small to large where the large cryogels and hydrogels in PVA had significantly lower swelling ratios than the small and medium shapes (*p* < 0.05), as shown in Figure 7b,d. Potential differences in the swelling kinetics could be attributed to slight differences during the scaffold fabrication. As the overall volume of the large mold is much different than that of the small, the freezing process may happen at a slightly different rate than in the small mold. The mold material may also influence the freezing time.

### 2.5. Ultimate Compression

**Figure 8:**
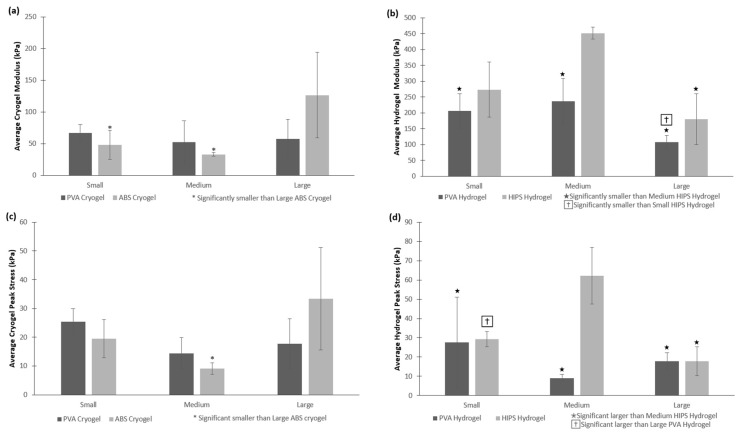
All cryogel and hydrogel scaffolds were compressed to 50%, providing both the average (**a**,**b**) modulus and (**c**,**d**) peak stress. None of the cryogels experienced any crack propagation, while most hydrogels experienced different levels of crack propagation. ★, †, and * denote significant differences (*p* < 0.05).

All cryogel and hydrogel scaffolds were subjected to 50% strain while in their hydrated state to determine their strength and durability. Note that the testing was completed in air and a biopsy punch of 6 mm, taken from the center of each scaffold, was used to perform these tests. Because of the complex shapes and dissolvent, there were some statistically significant differences in the peak stress and modulus of the scaffolds. The modulus of the large cryogels in ABS molds was significantly higher than any of the other cryogels, and the peak stress of the large cryogel in ABS molds was significantly higher than the medium sized cryogels in ABS molds, as shown in Figure 8a,c, respectively (*p* < 0.05). There were more significant differences for the peak stress and modulus between the hydrogels due to their brittleness, as shown in Figure 8b,d. However, most importantly, none of the cryogels showed any crack propagation despite being compressed by 50%, whereas most hydrogels experienced crack propagation after being compressed by 50% due to their brittle nature. The cryogels’ sponge-like structure and durability under compression makes this type of scaffold applicable for bone regeneration purposes.

### 2.6. Cellular Adhesion

**Figure 9:**
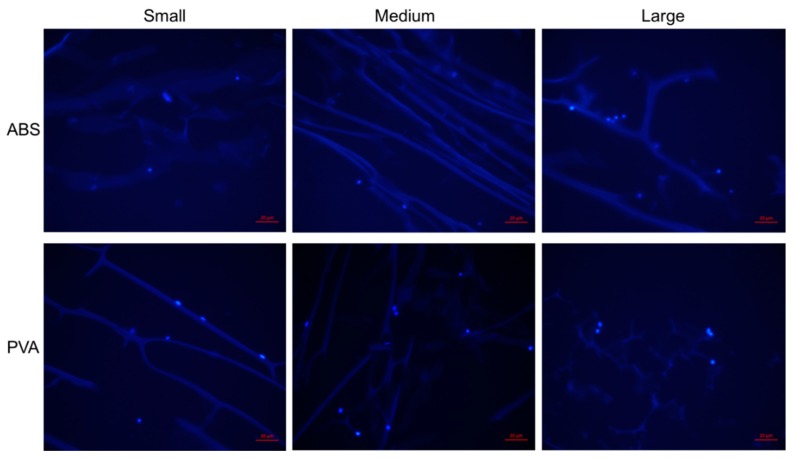
MG-63 cells were allowed to adhere on the cryogel scaffolds for 1 h. The cells’ nuclei can be visualized as the fluorescent blue dots. Cells were able to adhere on all scaffolds. The red scale bar denotes 20 µm.

Due to the immersion of cryogels in water and acetone for an extended period of time, the cryogels were assessed for initial biocompatibility by performing a cell adhesion test. As seen in Figure 9, MG-63 (osteoblast-like) cells were able to adhere on all scaffolds where the cells’ nuclei are shown as the lighter blue dots attached to the scaffolds’ pores. Despite the short incubation period, previous work has shown that such a protocol results in full infiltration of the CG cryogels after 28 days [16,22]. Visually it appears that cryogels in the PVA molds allowed more cells to adhere. Nonetheless, regardless of the geometry, mold, or dissolvent, cells were able to adhere on all cryogel scaffolds. Note that the cryosectioning process led to some shredding which resulted in the spread appearance of the cryogel struts, as seen within the figure.

### 2.7. Fit Test and Volume Comparison

**Figure 10:**
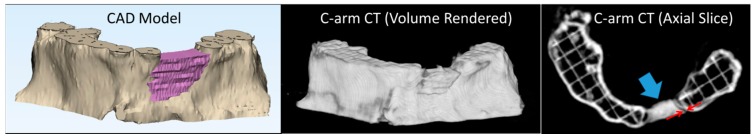
Computer-aided design (CAD) model of the defect site and the medium scaffold (**left**). Volume rendered C-arm CT scan of a medium cryogel scaffold placed in a 3DP defect site (**center**). One axial slice of the C-arm CT scan (**right**). The scaffold is indicated by the blue arrow and an example gap distance measurement is indicated by the red arrows.

The measurements for the fit test and volume comparison are shown in Table 1 and Table 2 for the cryogels and hydrogels, respectively. The cryogel and hydrogel scaffolds in their patient-specific shapes were placed in their respective defect sites in 3DP maxillas and then scanned using C-arm CT. The average maximum gap distance between the scaffold and the maxilla was determined for each image slice. An example C-arm CT scan, showing the medium scaffold placed in a 3DP defect site, along with the original CAD model, is shown in Figure 10.

The volume of each scaffold was measured and used to compute the percent difference between the volume of the mold and the actual volume of each scaffold. The 1D scale factor was also computed for each scaffold. While the volume differences (and 1D scale factors) between each of the mold sizes were statistically significant, the small and large volume differences were smaller and closer together than the medium mold. The volume differences of the cryogels were significantly smaller than their corresponding hydrogels. The gap distance between the medium and large hydrogel scaffolds was also significant.

## 3. Discussion

In this study, the combination of 3D printing and scaffold fabrication was used to create patient-specific cryogels and hydrogels for critical size craniofacial bone defects. This study is a continuation of our previous published article that provided a proof of concept analysis of cryogels in various desired shapes [16]. Having patient specific scaffolds would improve the surgical outcome and morbidity of patients with critical size defects. The goal of the study was to evaluate the best method to dissolve the 3DP molds while retaining both cryogel and hydrogel physical characteristics. Overall, all cryogels and hydrogels were able to properly form in the desired shaped of the mold. Fit test analysis provided quantitative data on the fit and shape of the scaffolds.

The SEM images show the morphology of the scaffolds. The rounded macropore morphology throughout all three shapes of the cryogels in ABS molds stayed consistent. Although the macropores on the cryogels in PVA molds seemed flaky, this did not affect its mechanical properties or cell compatibility. One possible reason the pores of the cryogels in the PVA molds appear different from the cryogels in the ABS molds may be due to the fact that PVA is hydrophilic, thus seeping through the cryogel and affecting the internal structure, whereas ABS is hydrophobic. Nonetheless, the flakiness did not appear to cause issues in cell adhesion and swelling potential. The hydrogels, however, did not present macropores thus making this scaffold not well suited for cell infiltration and angiogenesis.

The µCT analysis provided quantitative data of the pore structure of the cryogels. The data proved the similar pore structure between all cryogel scaffolds. The complex shapes, dissolvent, and mold material did not alter the internal pore structure of the cryogels. The size of the pore is important for the flow of nutrients and waste removal, as well as the infiltration of cells to be able to form the extracellular matrix and thus regenerate the bone, ideally the pore sizes for this to occur should be around 100 µm [34]. The pores in our patient-specific CG cryogels ranged between 26.6 and 28.6 µm. In a study by Kathuria et al., their CG cryogels’ pores were in the range of 30 to 50 µm [32]. Other CG cryogels fabricated previously in our lab have had pore sizes range between 20 to 40 µm, with the more complex shapes being on the lower end of the spectrum, but despite these smaller pore sizes cell infiltration was still possible [16,22]. Porosity tests were also performed to verify the void space in the cryogel scaffolds was ideal for cell infiltration and nutrient flow. The results obtained from this test can be compared to the results from the µCT analysis in Figure 5a. Although the µCT gave slightly lower values, they were still closely related, and most importantly, there were no significant differences between any of the cryogels. Furthermore, µCT measurements could be slightly inaccurate because it is done on hydrated scaffolds. Since we knew cell infiltration would be possible based on previous CG cryogel studies, our main concern in this study was the possible incompatible surface on the cryogels due to the mold material and dissolving solution. The biocompatibility was then verified in the cell adhesion test. However, while the pore sizes were slightly smaller than ideal, the data remained consistent throughout all cryogels.

Swelling tests are important to understand the scaffolds ability to swell and fill a defect site as well as absorb nutrients throughout the entire scaffold. It was shown that the cryogels were able to swell up to over 400% of their original dry weight within only 2 min of submersion, whereas the hydrogels’ swelling potential was much lower. This further demonstrates the advantages of using cryogels over hydrogels in regards to filling defect sites. The variance of the swelling potential in the scaffolds in the PVA molds could be due to the fact that the PVA seeps through the scaffolds during the dissolving process because of the hydrophilicity of the material, thus possibly affecting the cryogels’ ability to absorb water. Another possible reason could be the solution in which the molds dissolved in. Because the scaffolds are made of 99% water, there could have been a problem in the thawing and dissolving process for the PVA scaffolds, which dissolve in water, possibly affecting the pores. This can be visually represented in the SEM images. However, the cryogels in PVA molds were still able to swell up to over 400% of their original dry weight, verifying their capability to fill the defect site. Additionally, previous work has shown that larger and more complex molds can extend the freezing process, having an effect on the crosslinking and formation of the scaffold [16]. In this study, the volume of the large mold is much different than that of the small and thus, variations in fabrication could have occurred, affecting the scaffold’s structure and swelling potential. This can be seen with both the PVA and ABS cryogels. In comparison, the freezing process in not present for hydrogel fabrication and thus, the HIPS hydrogels did not experience this large difference between the various geometries. While the PVA hydrogels still displayed differences in swelling potential with the different size molds, this is most likely due to the PVA moving into the scaffold during dissolution, as described previously.

To further examine the mechanical properties of the scaffolds, the scaffolds were subjected to compression tests to 50% strain. As expected, none of the cryogels cracked under compression, whereas most hydrogels did. Because of the different sizes and complex shapes of the scaffolds, they may not have formed or frozen in the same manner, thus resulting in some significant differences in peak stress and moduli. Overall, the data ranges quite a lot where most HIPS hydrogels had higher peak stress and moduli than the PVA hydrogels. In comparison, the small and medium PVA cryogels had higher peak stress and modulus than the ABS cryogels; however, the large ABS cryogels had the higher values. Despite the cryogels having lower modulus than the hydrogels, these scaffolds were much more durable and did not fracture, as previously discussed. Previous studies completed in our lab have cyclically loaded CG cryogels, demonstrating their resilience and ability to withstand shape deformation over 28 days [22,33]. Additionally, CG hydrogels have also been shown to withstand similar cyclic loading conditions, resulting in slightly higher stress-relaxation and hysteresis values than the cryogels [33]. It should be noted that these scaffolds are not meant to replace bone, but rather provide a template for new tissue growth. Thus, it is not necessary that the scaffolds match the mechanical properties of bone, but rather provide a durable framework for cell infiltration and new bone formation. Future tests can include an increase in freezing time for the large cryogel, as well as achieving a more uniform punches in the middle of the scaffolds to minimize the differences.

To verify the materials used for the molds and dissolvent did not affect the biocompatibility of the scaffolds, a cell adhesion test was performed. Cells were able to adhere on all cryogels, regardless of the mold material, solution in which the cryogels dissolved in, as well as the complex shapes of the cryogels.

Overall the process of separating the scaffolds from the molds was much easier using the dissolving technique, as compared to the mechanical extraction technique used in our prior work [16]. The scaffold shapes were a more accurate representation of the molds, as there was no material left behind in the molds (as was the case for mechanical extraction). However, the mold dissolving technique caused a new issue, which was manifested in the volume comparisons shown in Table 1 and Table 2 for both cryogels and hydrogels. The measured scaffold volumes were smaller than the mold volumes. This issue is more pronounced for the hydrogels and also for the medium mold.

In our prior work, the scaffolds were removed from the molds and immediately scanned. For the current work, there was a delay of up to four hours, after the dissolving process was complete, before the scaffolds were scanned; time in which some minor dehydration and subsequent shrinkage could have occurred. This is a relatively minor effect in the small and large cryogels, which had average linear scaling factors of 0.95 and 0.9, respectively. The medium mold differs from the other two in several respects. The medium mold underwent less spatial filtering (as seen in Figure 1) and also contained some very thin sections. This mold also had the largest surface area to volume ratio. These differences could exacerbate both the cryogel’s smoothing of sharp mold edges, and any dehydration that may have occurred. With respect to the hydrogels, previous work has demonstrated their ability to both swell and shrink based on environmental factors including temperature [35,36]. Additionally, chitosan has previously been shown to shrink and deform following drying [37]. We hypothesize that, due to the delay in scanning, these factors caused the hydrogels to lose some water content, leading to their shrinkage.

Lastly, the images that were used to measure the gap distance were re-examined. It was found that the smaller scaffolds sat slightly lower in the defect volume, aided by gravity. This helped keep the gap distance low, even for smaller scaffold volumes. While investigation will continue into this scaffold volume issue, it is extremely easy to compensate for this effect in the 3-Matic (Research Version 11.0) software by adding a scaling factor (>1) to the molds to pre-compensate for any anticipated volume changes in the scaffold production process. For a given mold and scaffold material, the amount of reduction was consistent.

## 4. Materials and Methods

### 4.1. Creation of Patient Specific Degradable Molds

Anonymized computed tomography (CT) scans of three pediatric patients with craniofacial defects were obtained through an IRB waiver from Saint Louis University School of Medicine. The defects selected were those which would normally require a bone graft. The DICOM image data was first imported into Mimics (Research Version 19.0, Materialise, Leuven, Belgium). A bone threshold was used to isolate the maxilla, and the defect was segmented in a series of axial slices, creating a defect volume. This segmented volume was imported into 3-Matic (Research Version 11.0, Materialise, Leuven, Belgium) for further processing. After smoothing, a duplicate volume was created which had the same center, but was 2 mm larger in all three dimensions. The smaller volume was subtracted from the larger volume, resulting in a hollow mold that was approximately 1 mm thick, and in the shape of the defect volume. A filling hole was placed on the top of the mold. The dissolvable molds were then exported as STL files and printed on a Lulzbot TAZ 5 & 6 fused deposition modeling (FDM) printer (Aleph Objects, Loveland, CO, USA). The molds were printed using 3 mm ABS, PVA, and HIPS filament. All three mold materials were previously used on all scaffolds to determine the mold with the best outcome with regard to the handling and structure of the scaffolds. PVA and ABS were used to fabricate the cryogels and PVA and HIPS were used to fabricate the hydrogels.

### 4.2. Fabrication of CG Cryogels

Chitosan-gelatin (CG) cryogels were fabricated as previously described [32]. 10 mL of a 1% acetic acid (Fisher Scientific, Hampton, NH, USA). solution was made and 2 mL of this solution was set aside for later use. 80 mg of low viscosity chitosan (MP Biomedicals, Solon, OH, USA) was combined with the remaining 8 mL of the acetic acid solution. This combination was then mixed in a mechanical spinner for about 30 min or until all of the chitosan was dissolved. Once dissolved, 320 mg of gelatin from cold water fish skin (Sigma-Aldrich, St. Louis, MO, USA). was mixed into the chitosan solution and was placed on the spinner for another 30 min or until the gelatin was dissolved. A 1% glutaraldehyde (Sigma-Aldrich, St. Louis, MO, USA) solution was made by adding 1 µL of glutaraldehyde to the 2 mL acetic acid solution previously set aside. After the CG solution was thoroughly mixed, both the CG and glutaraldehyde solutions were placed in a 4 °C refrigerator for 1 h. After being cooled, both solutions were mixed by decanting between the vials. The mixed solution was then poured into the 3DP cooled degradable molds and immediately placed in a −20 °C freezer. The cryogels were then allowed to freeze for 24 h. After 24 h, the cryogels in their degradable molds were removed from the freezer and placed in their respected solutions to allow the cryogels to thaw and the molds to degrade, such that the molds disintegrated and separated from the scaffolds. The PVA molds were dissolved in deionized water for 2 h. The ABS molds were dissolved in acetone for 2 h. The HIPS molds were dissolved in Limonene and required 5 h.

### 4.3. Fabrication of CG Hydrogels

CG hydrogels were fabricated similarly to the cryogels. Briefly, 10 mL of a 1% acetic acid solution was made and 2 mL of this solution was set aside for later use. 80 mg of low viscosity chitosan was combined with the remaining 8 mL of the acetic acid solution. This combination was then mixed in a mechanical spinner for about 30 min or until all of the chitosan was dissolved. Once dissolved, 320 mg of gelatin from cold water fish skin was mixed into the chitosan solution and was on the spinner for another 30 min or until the gelatin was dissolved. A 1% glutaraldehyde solution was made by adding 1 µL of glutaraldehyde to the 2 mL acetic acid solution set aside from earlier. After the CG solution was thoroughly mixed, it was mixed with the glutaraldehyde solution by decanting between vials. This solution was poured into the 3DP degradable molds and left to gel at room temperature for 2 h. Following gelation, the hydrogels in their molds were placed in their respected solutions to allow the molds to degrade, as described in the previous section.

### 4.4. Pore Analysis (SEM and MicroCT)

Scanning electron microscopy (SEM; Zeiss, Evo LS15, Thornwood, NY, USA) was used to observe pore structure on the cryogels and hydrogels. After the scaffolds were removed from the degraded molds, they were frozen at −80 °C for 1 h. Afterwards, the scaffolds were lyophilized (Sentry 2.0 VirTis BenchTop Pro Freeze Dryer, SP Scientific, Warminster, PA, USA) for 24 h to remove all the water. The samples were then mounted on an aluminum stub and sputter coated (SoftComp, Bal-Tec SCD 005, Balzers, Liechtenstein) for 360 s in gold at 20 mA. The SEM (Zeiss, Evo LS15) was then used to obtain images at 200× and 500× for hydrogels and cryogels, respectively.

Microcomputed tomography (microCT) (mCT 35, Scanco Medical, Wayne, PA, USA) was used to obtain quantitative analysis of the cryogels by measuring pore sizes, interconnectivity, and the amount of polymer within the cryogels as previously described [33]. Note that this analysis method could only be completed for the cryogels due to the high fragility of the hydrogels. Three scaffolds (*n* = 3). from each mold shape were punched using a 5 mm biopsy punch to scan using the following parameters: projections 500, medium resolution scan, X-ray tube potential of 55 kVp, X-ray intensity of 4 W, isotropic voxel size of 7 microm, and integration time of 600 ms. By means of the manufacturer installed trabecular morphology analysis, the scaffold pore geometry and volume were quantified using a threshold of 80 per milles. Values above the threshold were considered scaffold while values under the threshold were considered empty space. The pore heterogeneity was quantified through the standard deviation of the pores, as output by the µCT.

### 4.5. Porosity Test

The porosity of the cryogels was measured using a method described by Garg et al. [38], based on Archimedes’ principle. Note that this was completed on only the cryogels as a comparison to the µCT data. Lyophilized cryogels (*n* = 3) were weighed and each immersed in 5 mL of ethanol (Fisher Scientific, Hampton, NH, USA) for 24 h. After 24 h of immersion, the cryogels were removed from ethanol, blotted on filter paper, and weighed once more. The change in weight of the scaffold is equivalent to the amount of ethanol present in the scaffold. The equation used to calculate percent porosity was

(1) $$\mathrm{Porosity}\left(\%\right)=\frac{V_{\mathrm{ETH}}}{V_{\mathrm{ETH}}+V_{\mathrm{CG}}},$$

where *V*_ETH_ is the volume of the intruded ethanol, calculated as the ratio of the observed mass change after intrusion and density of ethanol (*ρ*_ETH_ = 0.789 g/mL), and *V*_CG_ is the volume of the cryogel samples, calculated as the ratio of the dry scaffold mass and density of the CG cryogel (*ρ*_CG_ = 0.682 g/mL).

### 4.6. Swelling Kinetics

Lyophilized samples (*n* = 3) were weighed before being submerged in water to get the dry weight. After weighing, the scaffolds were submerged in water and the weight was taken after 2, 4, 10, 20, 40 min, 1, 2, 4, and 24 h. The swelling ratio was then determined by using the equation

(2) $$\mathrm{Swelling}\;\mathrm{Ratio}=\left(W_{s}-W_{d}\right)/W_{d},$$

where *W*_s_ is the scaffold’s wet weight and *W*_d_ is the scaffold’s dry weight [33].

### 4.7. Ultimate Compression

Compression tests were conducted for each scaffold and shape (*n* = 3) at 50% strain. A 6-mm biopsy punch (Acuderm, Inc., Fort Lauderdale, FL, USA) was used to obtain similar diameters of each scaffold. The thickness and diameter of each sample was measured and recorded. The mechanical testing system (MTS Criterion Model 42, MTS Systems Corporation, Eden Prairie, MN, USA) used a 100 N load cell with the following parameters: data acquisition of 10 Hz, strain rate of 10 mm/min, preload of 0.05 N, and preload speed of 1 mm/min. Peak stress (kPa) and Young’s modulus (kPa) were obtained from MTS TW Elite software.

### 4.8. Cellular Adhesion

A cellular adhesion test was performed to verify the mold material and dissolvent did not affect the surface of the cryogels. Note that this was only completed on cryogels due to their macroporous structure, a property ideal for cell infiltration and angiogenesis. The cryogels were cut as previously described to have similar size and shape. Cryogel samples (*n* = 3) of the three mold geometries in PVA and ABS were sterilized by soaking them in 70% ethanol (Fisher Scientific, Hampton, NH, USA) for 30 min and then rinsing them with sterile phosphate-buffered saline (PBS; GE Healthcare Biosciences, Pittsburgh, PA, USA) three times for 10 min. After sterilization, the cryogel samples were placed onto a sterile 36-well plate and seeded with 100,000 human-bone osteosarcoma-derived cells (MG-63, passage 4; ATCC, Manassas, VA, USA) in 100 µL of media. The media was composed of dulbecco’s modified eagle’s medium (DMEM) with 4.5 g/L glucose and L-glutamine (Lonza, Walkersville, MD, USA), 10% fetal bovine serum (FBS, Biowest, San Marcos, TX, USA) and 1% penicillin-streptomycin solution (Hyclone, Pittsburgh, PA, USA). The samples were then incubated for 1 h at 37 °C and 5% CO_2_. Afterwards, an additional 300 µL of media was added to the scaffolds and placed again in the incubator for 1 h at 37 °C and 5% CO_2_. The media was then removed and the cryogels were placed onto a new well plate. They were then rinsed twice with sterile PBS for 5 min each on a shakerplate. The cryogel samples were then immediately placed in formalin (Fisher Healthcare, Hampton, NH, USA) for 24 h for sectioning. After 24 h, the cryogel samples were then placed in a 30% sucrose (Acros, Livingston, NJ, USA) solution for an additional 24 h and then embedded in optimal cutting temperature (OCT, Fisher Healthcare, Hampton, NH, USA) overnight. The cryogels were then cryosectioned at 20 µm and stained with 4′,6-diamidino-2-phenylin- dole (DAPI, Acros, Livingston, NJ, USA) to assess cellular adhesion.

### 4.9. Accuracy of Fit and Scaffold Volume

After the molds were dissolved in their respective solutions, all PVA cryogels and hydrogels for all three defects were scanned in their respective 3DP maxilla. Note that PVA was chosen for this final analysis based on ease of handling and previous data. C-arm CT scans (20-s neuro protocol) performed on a neuro-angiography system (Siemens, Erlangen, Germany) were used to measure the fit of the cryogel in the 3DP maxilla, and for volume comparison. The DICOM files collected for each scan were imported to Mimics (Research Version 19.0, Materialise, Leuven, Belgium) for analysis. The volumes were rendered by thresholding the pixels (of the axial slices) within the Hounsfield Unit range of the scaffold. The volume difference was computed as

(3) $$\mathrm{Volume}\;\mathrm{Difference}=\frac{V_{m}-V_{s}}{V_{m}},$$

where *V*_m_ is the volume of the mold, and *V*_s_ is the volume of the sample.

A one-dimensional scale factor was also computed from the measured volume and mold volume as

(4) $$1D\;\mathrm{Scale}\;\mathrm{Factor}=\sqrt[{3}]{\frac{V_{s}}{V_{m}}}.$$

This factor characterizes the volume difference in terms of the average one-dimensional (1D) change in the scaffold size that resulted in the measured volume difference. The average maximum gap distance between the mold and the jaw was determined by measuring the largest gap distance from each slice and computing the mean.

### 4.10. Statistical Analysis

Statistical analysis was done with SPSS software (IBM) by means of appropriate ANOVA with a Tukey post-hoc analysis. The alpha value was set at 0.05 for statistical significance.

## 5. Conclusions

This study demonstrated that we were able to fabricate cryogel and hydrogel scaffolds in 3D printed patient specific craniofacial defect geometries using dissolvable mold materials. Having dissolvable mold materials in patient specific geometries can alleviate post-fabrication deformation of the cryogels. Cryogels possessed the desired swelling kinetics, swelling from 400% to almost 1500% of their original dry weight, which is important to fill the defect site and absorb nutrients throughout the entire scaffold. Cryogels also showed sponge-like consistency and mechanical durability during mechanical testing, as well as good biocompatibility. Additionally, pore sizes ranging from 26.6 to 28.6 µm and porosity from 79.7% to 87.2% were consistent throughout the cryogel scaffolds, regardless of geometry and mold material. The hydrogel scaffolds demonstrated that their nanoporous and brittle structure was not ideal for critical size bone regeneration, however they were still able to be formed in the desired shape. While their ability to fill the defect site in the 3DP maxillas was not ideal, the molds can be fabricated in a larger size. These characterization tests demonstrate that despite the complex geometries as well as mold material and solution, cryogel and hydrogel scaffolds maintained their properties.

## Figures and Tables

**Table 1:** Cryogel scaffold fit test and volume comparison analysis. * denotes significant differences between one mold and the other two, and † denotes a significant difference between a cryogel and its corresponding hydrogel (*p* < 0.05).

Cryogels	Small			Medium			Large		
	Avg Max Gap Dist (mm)	Volume Diff (%)	1-D Scale Factor	Avg Max Gap Dist (mm)	Volume Diff (%)	1-D Scale Factor	Avg Max Gap Dist (mm)	Volume Diff (%)	1-D Scale Factor
Trial 1	0.62	18.6	0.93	0.73	42.8	0.83	0.71	28.1	0.90
Trial 2	0.66	7.70	0.97	0.74	42.5	0.83	0.77	23.8	0.91
Trial 3	0.54	12.8	0.96	0.66	40.6	0.84	0.89	30.7	0.88
Average	0.61	13.0 *^,†^	0.95 *^,†^	0.71	41.9 *^,†^	0.83 *^,†^	0.79	27.5 *^,†^	0.90 *^,†^

**Table 2:** Hydrogel scaffold fit test and volume comparison analysis. * denotes significant differences between one mold and the other two, † denotes a significant difference between a hydrogel and its corresponding cryogel, and ★ denotes a significant difference in average maximum gap distance (*p* < 0.05).

Hydrogels	Small			Medium			Large		
	Avg Max Gap Dist (mm)	Volume Diff (%)	1-D Scale Factor	Avg Max Gap Dist (mm)	Volume Diff (%)	1-D Scale Factor	Avg Max Gap Dist (mm)	Volume Diff (%)	1-D Scale Factor
Trial 1	0.80	37.4	0.86	1.00	56.7	0.76	0.49	3.5	0.83
Trial 2	0.71	41.1	0.84	0.91	50.6	0.79	0.60	42.1	0.83
Trial 3	0.51	38.4	0.85	0.79	50.3	0.79	0.42	44.5	0.82
Average	0.67	39.0 *^,†^	0.85 *^,†^	0.90 ^★^	52.5 *^,†^	0.78 *^,†^	0.50 ^★^	43.4 *^,†^	0.83 *^,†^
